# Correction: Chisholm et al. Frontline and Relapsed Rhabdomyosarcoma (FaR-RMS) Clinical Trial: A Report from the European Paediatric Soft Tissue Sarcoma Study Group (EpSSG). *Cancers* 2024, *16*, 998

**DOI:** 10.3390/cancers16193427

**Published:** 2024-10-09

**Authors:** Julia Chisholm, Henry Mandeville, Madeleine Adams, Veronique Minard-Collin, Timothy Rogers, Anna Kelsey, Janet Shipley, Rick R. van Rijn, Isabelle de Vries, Roelof van Ewijk, Bart de Keizer, Susanne A. Gatz, Michela Casanova, Lisa Lyngsie Hjalgrim, Charlotte Firth, Keith Wheatley, Pamela Kearns, Wenyu Liu, Amanda Kirkham, Helen Rees, Gianni Bisogno, Ajla Wasti, Sara Wakeling, Delphine Heenen, Deborah A. Tweddle, Johannes H. M. Merks, Meriel Jenney

**Affiliations:** 1 Children and Young People’s Unit, Royal Marsden Hospital and Institute of Cancer Research, Sutton SM2 5PT, UK; henry.mandeville@rmh.nhs.uk; 2 Children’s Hospital for Wales, Cardiff CF14 4XW, UK; madeleine.adams@wales.nhs.uk (M.A.); meriel.jenney@wales.nhs.uk (M.J.); 3 Department of Paediatric Oncology, Gustave Roussy, 75015 Paris, France; veronique.minard@gustaveroussy.fr; 4 Department of Paediatric Surgery, University Hospitals Bristol and Weston NHS Foundation Trust, Bristol BS1 3NU, UK; timothy.rogers@uhbw.nhs.uk; 5 Department of Paediatric Histopathology, Royal Manchester Children’s Hospital, Manchester University NHS Foundation Trust, Manchester M13 9WL, UK; anna.kelsey@mft.nhs.uk; 6 The Institute of Cancer Research, London SW7 3RP, UK; janet.shipley@icr.ac.uk (J.S.); ajla.wasti@rmh.nhs.uk (A.W.); 7 Department of Radiology and Nuclear Medicine, University of Amsterdam, Amsterdam UMC, 1081 HV Amsterdam, The Netherlands; r.r.vanrijn@amsterdamumc.nl; 8 Princess Máxima Center for Pediatric Oncology, 3584 CS Utrecht, The Netherlands; i.s.a.devries-10@prinsesmaximacentrum.nl (I.d.V.); r.vanewijk-2@prinsesmaximacentrum.nl (R.v.E.); b.dekeizer@prinsesmaximacentrum.nl (B.d.K.); j.h.m.merks@prinsesmaximacentrum.nl (J.H.M.M.); 9 Birmingham Women’s and Children’s NHS Foundation Trust, Birmingham B15 2TG, UK; s.gatz@bham.ac.uk; 10 Cancer Research UK Clinical Trials Unit, Institute of Cancer and Genomic Sciences, University of Birmingham, Birmingham B15 2TT, UK; c.m.firth@bham.ac.uk (C.F.); k.wheatley@bham.ac.uk (K.W.); p.r.kearns@bham.ac.uk (P.K.); wenyu.liu@ndph.ox.ac.uk (W.L.); a.j.kirkham@bham.ac.uk (A.K.); 11 Fondazione IRCCS Istituto Nazionale Tumori, 20133 Milan, Italy; michela.casanova@istitutotumori.mi.it; 12 University Hospital Copenhagen, DK-2200 Copenhagen, Denmark; lisa.lyngsie.hjalgrim@regionh.dk; 13 Department of Paediatric Oncology, University Hospitals Bristol and Weston NHS Foundation Trust, Bristol BS1 3NU, UK; helen.rees@ubhw.nhs.uk; 14 Department of Women and Children’s Health, University of Padova, 35122 Padua, Italy; gianni.bisogno@unipd.it; 15 Alice’s Arc Charity, London EC1V 1AW, UK; sara.wakeling@alicesarc.org; 16 KickCancer Foundation, 1000 Brussels, Belgium; delphine@kickcancer.org; 17 Vivo Biobank, Translational & Clinical Research Institute, Newcastle University Centre for Cancer, Newcastle University, Newcastle upon Tyne NE1 7RU, UK; deborah.tweddle@newcastle.ac.uk

The authors wish to make corrections to the authorship and title of [1]. The corrected authorship is as follows: Julia Chisholm and Henry Mandeville contributed equally to the first authorship. Johannes H. M. Merks and Meriel Jenney contributed equally to senior authorship. In the title FAR-RMS has been corrected to FaR-RMS.

The authors apologize for any inconvenience caused and state that the scientific conclusions are unaffected. This correction was approved by the Academic Editor. The original publication has also been updated.
